# Relationship between poverty and symptoms of depression and anxiety among adolescents in Nepal: Examining the mediating effect of external and internal resilience

**DOI:** 10.1016/j.jad.2025.120589

**Published:** 2025-11-01

**Authors:** Rakesh Singh, Parinati Khanal, Emily Garman, Wietse A. Tol, Philip Jefferies, Mark J.D. Jordans, Crick Lund

**Affiliations:** a Centre for Global Mental Health, Health Service and Population Research Department, Institute of Psychiatry, Psychology and Neuroscience, King’s College London, London, UK; b Research Department, Transcultural Psychosocial Organization Nepal, Baluwatar, Kathmandu, Nepal; c Alan J Flisher Centre for Public Mental Health, Department of Psychiatry and Mental Health, University of Cape Town, Cape Town, South Africa; d Department of Public Health, Section for Global Health, University of Copenhagen, Copenhagen, Denmark; e VU University Amsterdam, Amsterdam, the Netherlands; f Atlantic Technological University, Castlebar, Mayo, Ireland

**Keywords:** Anxiety, Depression, Resilience, Mediation, Poverty, Adolescents

## Abstract

**Objective:**

This study aimed to examine the mediating effect of two types of resilience (internal, with factors such as problem solving and perseverance, and external resilience, with factors such as social support and sense of belonging) among adolescents affected by family poverty in Nepal in relation to symptoms of depression and anxiety.

**Methods:**

Participants identified as living in deprived conditions (*n* = 491) in a cross-sectional survey completed measures about poverty, depressive and anxiety symptoms, and external and internal resilience. Mediation analyses were performed using poverty scores as the predictor, internal and external resilience as mediators, and depression and anxiety scores as outcomes, tested through parallel and serial mediation models.

**Results:**

External resilience emerged as the only pathway across both parallel and serial mediation models. External resilience showed significant indirect effects for both depression (β = 0.797, 95 % CI: 0.181–1.677) and anxiety (β = 0.557, 95 % CI: 0.101–1.237). No sequential mediation was found. Although the total association of poverty with depression and anxiety was not significant—likely due to participants’ homogeneity in poverty—external resilience accounted for 54.33 % of the association with depression and 31.61 % with anxiety, contributing to total indirect effects of 66.79 % and 38.25 %, respectively.

**Conclusions:**

This study expands the evidence on the mediating effect of external resilience in the association between poverty and depression and anxiety in Nepali adolescents. The mediating effect was stronger for depressive outcomes than for anxiety. Factors of external resilience are discussed as a critical target for mental health interventions among adolescents living in poverty.

## 1 Introduction

Adolescence is a critical developmental stage marked by significant biological, psychological, and social changes that increase the global risk of mental health issues (Lin and Guo, 2024). Hormonal fluctuations and brain development during this phase can lead to mood swings and behavioral changes, making adolescents more vulnerable to mental health conditions, including depression and anxiety (Blakemore et al., 2010). Globally, around one in seven adolescents aged 10–19 has a mental health condition, contributing approximately 15 % of the disease burden in this age group (World Health Organization, 2024). Mental health conditions affect about 166 million adolescents, with anxiety and depression accounting for around 40 % of these conditions (UNICEF, 2021). Depression and anxiety are the most prevalent mental health conditions during adolescence, contributing significantly to disability and premature mortality (Erskine et al., 2015). Given that 90 % of the total child and adolescent population lives in low- and middle-income countries (LMIC) (Kieling et al., 2011), 80 % of the global burden of common mental disorders, including depression and anxiety, resides in LMICs (World Health Organization, 2018; Mudunna et al., 2025).

Poverty is a major factor that increases the risk of mental health problems (Thapar et al., 2017), including common mental disorders such as depression and anxiety (Lund et al., 2010; Patel et al., 1999; Patel and Kleinman, 2003). Multidimensional poverty—defined as deprivation across various dimensions such as education, health, and living standards—has been increasingly recognized as a critical determinant of adolescent mental health outcomes (Díaz et al., 2022). Research shows that children raised in families facing persistent poverty, especially during adolescence, are more likely to experience anxiety and depression, with lasting effects into adulthood (Najman et al., 2010; Ridley et al., 2020).

Nepal is one of the poorest countries in the South Asian region, with a poverty rate of 20.1 % (United Nations Development Programme, 2024). While research remains limited, existing evidence suggests that Nepali adolescents face a heightened risk of depression and anxiety. Available studies show a wide variation in the prevalence of depression, ranging from 5.2 % to 56.5 % (Silwal et al., 2018; Sharma and Kar, 2019; Nepal Health Research Council, 2021; Paudel et al., 2020; Gautam et al., 2021; Karki et al., 2022) and the prevalence of anxiety, ranging from 8 % to 55.6 % (Dangal and Bajracharya, 2020; Paudel et al., 2020; Karki et al., 2022; Ojha et al., 2013). The wide variation may be due to the range of tools used to measure depression and anxiety, differences in study locations, such as rural versus urban areas, and smaller sample sizes (Steel et al., 2009).

Fortunately, not all adolescents who come from a low socioeconomic background or who experience poverty-related stressors develop mental health problems. Research has focused on processes of resilience that can explain how adolescents exposed to adversity retain and regain wellbeing. Modern resilience theory highlights that resilience arises from both internal and external protective factors (Tomko et al., 2022), shaped by ongoing interactions between individuals and their environments (Betancourt and Khan, 2008; Ungar, 2011). The concept of multisystemic resilience highlights protective factors and processes operating across biological, psychological, and social/environmental levels—essentially reflecting Bronfenbrenner’s ecological model—and can be more simply operationalized as internal and external protective factors (Ungar et al., 2021). Resilience processes may include self-regulation, social support, attachment, and cultural traditions (Masten and Wright, 2010).

Internal protective factors may include ability, skills, or psychological characteristics such as help-seeking, optimism, self-efficacy, active coping, perseverance, hardiness, self-belief, religious beliefs, and positive thinking (Liu et al., 2022; Sousa et al., 2013; Tomko et al., 2022; Jefferies et al., 2022). External protective factors refer to situational supports outside the individual, such as supportive relationships, beneficial environmental organization, and access to community resources (Pangallo et al., 2015). During resilience processes, similar to protective factors, the risk factors would also come into play, for instance, while internal protective factors counteract risk factors such as stress, and the risk exposure can build coping skills. Expanding on these ecological frameworks, resilience in adolescence therefore involves managing risk and protection against depression and anxiety through a combination of internal factors such as optimism, problem-solving, purpose, and perseverance (Hielscher et al., 2023; Michelson et al., 2022; Patton et al., 2011; Ruan et al., 2022; Van Doren et al., 2019)— and external resources such as supportive family, peers, schools, and broader social networks (Mesman et al., 2021; Lane et al., 2025). Even among high-risk adolescents, access to such resources can foster coping and mental well-being well into adulthood (Zimmerman and Brenner, 2010).

Although resilience is well-documented as a buffer against the impact of poverty on adolescent mental health in high-income countries (HIC), there is limited research on its mediating role in low- and middle-income countries (LMIC). In contexts like Nepal, adolescents face different realities than adolescents in HIC. These include a more collectivist orientation, the availability of traditional family, community, and religious social support systems, weaker government institutions (including limited formal mental health services), and a rich range of cultural understandings of family and resilience. Moreover, LMICs encounter unique contextual challenges, including higher poverty rates, under-resourced health systems, that may influence the pathways through which poverty affects mental health. There remains weak empirical evidence on whether resilience mediates the relationship between poverty and adolescent mental health outcomes in LMICs, including Nepal. Understanding these mediating pathways could offer valuable insights for intervention strategies and policy development in Nepal’s adolescent mental health sector.

This study used the socio-ecological framework and the stress-buffering framework to explore the mediational role of resilience (both internal and external) in the relationship between poverty and depressive and anxiety symptoms among adolescents in Nepal.

## 2 Methods

### 2.1 Study design, setting, participants, and procedures

This cross-sectional study was embedded within a larger research project, *Improving Adolescent Mental Health by Reducing the Impact of Poverty* (*ALIVE*), which aims to develop and evaluate an intervention that addresses multidimensional poverty and self-regulation to prevent depression and anxiety among adolescents living in urban poverty (Lund et al., 2023). The ALIVE project is currently being conducted and has several sub-studies, including a formative study, a tool adaptation study, a cross-sectional survey, a pilot trial, and an adolescent engagement study (Lund et al., 2023). The resilience measures were included in the ALIVE cross-sectional survey in Nepal, specifically for the purpose of this study. The current study (ALIVE cross-sectional survey) was conducted among adolescents aged 13–15 years enrolled in public secondary schools of Budhanilkantha municipality and its adjacent wards of Kathmandu metropolitan city (wards 6 and 7). These areas were selected for their high vulnerability to poverty, aligning with ALIVE’s focus on urban poverty, dense location, and logistical feasibility for school-based recruitment. Of the 21 public secondary schools in the study area, 11 were eligible with the inclusion criteria for the public schools requiring at least 100 adolescent students across grades 6–8 and proximity between two schools (>1 km). Eight eligible schools were randomly chosen by the statistician (EG) for the survey, considering adequate to meet the required sample size for the larger ALIVE project (Lund et al., 2023). We used the term ‘adolescents’ rather than ‘middle school students’ in this study because, in Nepal, the grades (6–8, ages 13–15) fall under secondary school, and the study specifically targets this developmental stage when depression and anxiety symptoms typically emerge. The sample size for this cross-sectional survey was determined to meet the requirements of the ALIVE pilot Randomized Controlled Trial, for which this survey served as a sub-study. The pilot trial required 240 participants, based on recommendations for estimating key design parameters in pilot studies (Teare et al., 2014; Jordans et al., under review). To ensure that sufficient participants would be eligible for the pilot trial (screened negative for depression and anxiety), we targeted a survey sample of approximately 500 adolescents screening positive for poverty. To confirm that this sample size was also adequate for descriptive analyses in the current study, we ran post-hoc power analyses. Assuming a prevalence of 21.2 % for anxiety symptoms (Ghimire et al., 2024), a 95 % confidence level, a margin of error of ±4 %, and a 10 % non-response rate, the minimum required sample size was 447 participants. Additionally, although no a priori sample size calculation was conducted for mediation analyses, post-hoc power estimation indicated that the observed sample of 490 adolescents provided approximately 80 % power to detect the significant indirect effect of poverty on depressive symptoms through external resilience (CYRM-R; β = 0.797, SE = 0.384) at α = 0.05 (two-tailed), based on a *Z*-test approximation of the indirect effect (Fritz and MacKinnon, 2007). A total of 635 adolescents were recruited from eight schools for poverty screening, ensuring proportional distribution across schools, gender, age (13–15 years), and grades (6–8), with at least 50 participants per school. This number (*N* = 635) represents the total pool of students within the targeted grades (6 to 8) and age range (13–15 years) who were available and consented to participate at the time of recruitment. We assumed that 80 % of the participants would meet the poverty screening criteria to enable us to reach our targeted sample size of around 500 adolescents. A minimum of 50 participants per school was targeted, based on the total number of eligible students available in the smallest selected school, to ensure adequate representation across schools. Between February and March 2024, one-to-one face-to-face interviews (in Nepali) were conducted with the research participants by research assistants whom the lead author trained, particularly on research methods, ethics, consent and assent, adverse event reporting and management, interviewing, and the Open Data Kit (ODK) data collection process and storage. Data were recorded in the ODK platform using Android tablets. Out of 635 participants, 491 scored a deprivation index of ≥0.33 on the eight-item poverty screener tool developed for the study (see below), and were eligible for the study and completed the full survey. One of the adolescents dropped out, and the remaining 490 adolescents were included in the analysis.

### 2.2 Measures

#### 2.2.1 Poverty assessment

The Multi-Dimensional Poverty Index was adapted into an eight-item screening tool as part of the ALIVE study to identify adolescents living in multidimensional poverty (Lund et al., 2023) in the local context, drawing from indicators used in national surveys. The tool assessed factors such as regular family income, income level relative to the national poverty line, dependency ratio, education levels of the household head and other family members, access to a private toilet and kitchen, and ownership of key physical assets. Each item was assigned a specific weight (1/9 for each of the three items related to income and dependency, 1/6 for each of the two items related to education, and 1/9 for each of the three items related to access to sanitation and physical assets), and a total deprivation score was calculated. Adolescents with a score of 0.33 or higher were classified as living in poverty. While no formal psychometric assessments have been done, the poverty instrument was developed through expert consultations and piloted during the ALIVE tool adaptation phase to ensure contextual relevance and comprehension. For example, preliminary validation in Nepal showed that among 67 participants included in the pretesting, 62 screened positive for poverty, and subsequent qualitative assessments supported the tool’s contextual and construct relevance for identifying individuals living in poverty.

#### 2.2.2 Depressive symptoms

The Patient Health Questionnaire adolescent version - PHQA (Luitel et al., 2024), as a part of the Measure of Mental Health among Adolescents and Young People at the Population Level (MMAPP) tool (Carvajal-Velez et al., 2023); was used to assess the depressive symptoms. The higher the PHQA total score, the higher the depressive symptoms. The Cronbach’s alpha was 0.79 for the PHQA in this study.

#### 2.2.3 Anxiety symptoms

The Generalized Anxiety Disorder-GAD7 (Luitel et al., 2024), as a part of the MMAPP tool, was used to assess anxiety symptoms. The higher the GAD-7 total score, the higher the anxiety symptoms. The Cronbach’s alpha was 0.79 for the GAD-7 in this study.

#### 2.2.4 Resilience

Resilience develops from both internal and external protective factors by facilitating positive adaptation to adverse conditions or stressors. External protective factors refer to protective resources and supports that exist outside the individual, such as interpersonal skills and supportive relationships (Pangallo et al., 2015). Internal protective factors include intrapersonal capacities or psychological characteristics such as help-seeking, optimism, and self-efficacy (Liu et al., 2022; Sousa et al., 2013; Tomko et al., 2022; Jefferies et al., 2022). This distinction aligns with resilience theory, which differentiates between individual traits and the social-ecological supports that facilitate positive adaptation to adversity. In this study, resilience is examined as external resilience (encompassing external protective factors) and internal resilience (focusing on internal protective factors).

##### 2.2.4.1 External resilience

The 17-item Child and Youth Resilience Measure-Revised (CYRMR) (Jefferies et al., 2019) was used to assess external resilience, with preliminary validation in Nepal (Singh et al., 2025, under review). Aligning with the socioecological framework, the CYRMR consists of items that tap external protective resource factors, such as sense of belonging to the broader environment, availability and quality of support from caregivers, interpersonal skills, peer support, and social skills. The higher scores indicated higher external resilience. In the current study, sufficiently high internal consistency was found (α = 0.84).

##### 2.2.4.2 Internal resilience

The 10-item Rugged Resilience Measure (RRM) (Jefferies et al., 2022) was used to measure internal resilience, with preliminary validation in Nepal (Singh et al., 2025, under review). The RRM consists of protective factors commonly implicated in internal resilience, including the ability to cope with stress, adaptability, emotional self-regulation, meaning-making/purpose, motivation/embracing challenges, optimism, perseverance, pride in achievements, problem-solving ability, and self-belief. Greater scores indicated greater internal resilience. In the current study, sufficiently high internal consistency was found (α = 0.82).

#### 2.2.5 Data analysis

Descriptive statistics such as frequency, percentage, mean, and standard deviation were explored to describe participant characteristics. Assumptions of normality for all continuous variables were confirmed through histograms and Q-Q plots, and linearity, homoscedasticity, and residuals normality were confirmed using scatterplots, histograms of residuals, and P-P plots. Bivariate correlations using Pearson’s correlation or point-biserial correlations were explored for continuous variables, and multicollinearity diagnostics using variance inflation factor and Tolerance were confirmed. We found that there were no problematic multicollinearity issues before running the mediation analysis. Additionally, we also performed an exploratory bivariate correlation between poverty and levels of depressive and anxiety symptoms (see Supplementary File 1). Depression categories were defined as follows: no/minimal symptoms (PHQ-A score 0–4), mild (5–9), moderate (10–14), moderately severe (15–19), and severe (≥20) (Kroenke et al., 2001). Anxiety categories were similarly defined: no/minimal symptoms (GAD-7 score 0–4), mild (5–9), moderate (10–14), and severe (≥15) (Spitzer et al., 2006).

Parallel mediation analysis was conducted using the PROCESS macro (Model 4) in SPSS v29 (IBM Corp., 2022). Bootstrap resampling (5000 samples) was used to estimate bootstrap confidence intervals for indirect effects. Significance was set at *p* < 0.05, and 95 % Confidence Intervals (CI) were estimated to assess direct, total, and indirect effects. In the parallel mediational model, the poverty score was treated as the predictor. Depressive and anxiety symptoms (as a continuous total score of PHQA and GAD7) of adolescents were treated as two outcomes in separate models. External resilience (CYRMR total score) and internal resilience (RRM total score) were treated as separate mediators in the parallel mediation models. Gender was included as a covariate in the models due to existing evidence of higher anxiety and depression rates among females (Shawon et al., 2024; Giri et al., 2024). The percentage of the total effect accounted for by the indirect (mediating) effect was also computed (Pieters, 2017; Shrout and Bolger, 2002). According to the guidelines by Preacher and Hayes (2004, 2008), mediation is established when the indirect effect is statistically significant and its bootstrapped CI does not include zero. The modern perspective was used to perform mediation analysis, considering that a significant association between an independent variable and dependent variable is not a prerequisite for examining the indirect effect of the independent variable on the dependent variable through the mediator (Hayes, 2009; Zhao et al., 2010). The statistical analysis plan was pre-registered before analysis on Open Science Framework (OSF) (https://doi.org/10.17605/OSF.IO/E2P5U). While our original statistical analysis plan, pre-registered on the OSF, included only parallel mediation models to assess the independent indirect effects of internal resilience and external resilience in the relationship between poverty and depressive and anxiety symptoms, we conducted an additional exploratory serial mediation analysis to explore the potential interrelationships between the mediators (external and internal resilience) in the model by testing whether the effect of poverty on depression/anxiety symptoms operates through a causal chain linking external resilience and internal resilience sequentially (i. e., poverty → external resilience → internal resilience → depression/anxiety symptoms; and poverty → internal resilience → external resilience → depression/anxiety symptoms). The rationale for this serial mediation model is grounded in the theoretical argument, such as socioecological resilience theory, noting that resilience depends more on what individuals can access than what they have within (Ungar, 2011), suggesting that external protective factors (external resilience) may be necessary to foster internal protective factors (internal resilience), or vice versa. Therefore, we applied the PROCESS macro (Model 6) to examine possible serial mediation pathways. Bootstrapped confidence intervals (5000 resamples) were used to assess the significance of indirect effects.

### 2.4 Ethics

Ethics approval was obtained from the ethics committee of King’s College London (HR/DP-23/24-38680; RESCM-23/24-386w80) and Nepal Health Research Council (ref. no. 896). Data collection permission was granted by municipal education units and school principals. Informed written assent and consent were obtained from the research participants and their caregivers before their enrollment in the study.

## 3 Results

### 3.1 Participant characteristics

The mean age of the participants was 13.85 (SD = 0.73), with females comprising 52.7 %. The majority were *Janajati* (50.4 %) by ethnicity (traditionally underrepresented ethnicities), and most were enrolled in 7th grade (42.9 %) (see Table 1).

### 3.2 Correlations between measures

The bivariate correlations (see Table 2) indicate that poverty scores were positively correlated with anxiety scores (*r* = 0.095). Both poverty and mental health outcomes, including depression and anxiety scores, were negatively correlated with both external and internal resilience scores. Depression and anxiety scores were positively correlated (*r* = 0.779). The correlation between external and internal resilience scores was significantly positive (*r* = 0.656); however, the variance inflation factor was 1.757, and the tolerance was 0.569, indicating no multicollinearity issues between CYRMR and RRM. These were therefore treated as two separate variables (mediators) in the proposed parallel mediation analyses. Gender was positively correlated with depression and anxiety scores and negatively correlated with internal resilience scores.

The findings of the exploratory sub-group analysis related to bivariate correlations between poverty and different levels of depression and anxiety symptoms (see Table S1, Supplementary File 1) revealed that only mild depression showed a small but significant negative correlation with poverty (*r* = −0.139, 95 % CI −0.270, −0.002), while all other groups had inconsistent directions and non-significant correlations—likely reflecting the homogeneously poor sample—no clear pattern of association across symptom categories emerged.

### 3.3 The mediational effect of resilience on depressive and anxiety symptoms

Two parallel mediation models were analyzed: 1) to examine whether the relationship between poverty and *depressive* symptoms was mediated by external resilience (CYRMR) and internal resilience (RRM), 2) to examine whether the relationship between poverty and *anxiety* symptoms was mediated by external resilience and internal resilience. In both models, we controlled for gender.

**Fig. 1:**
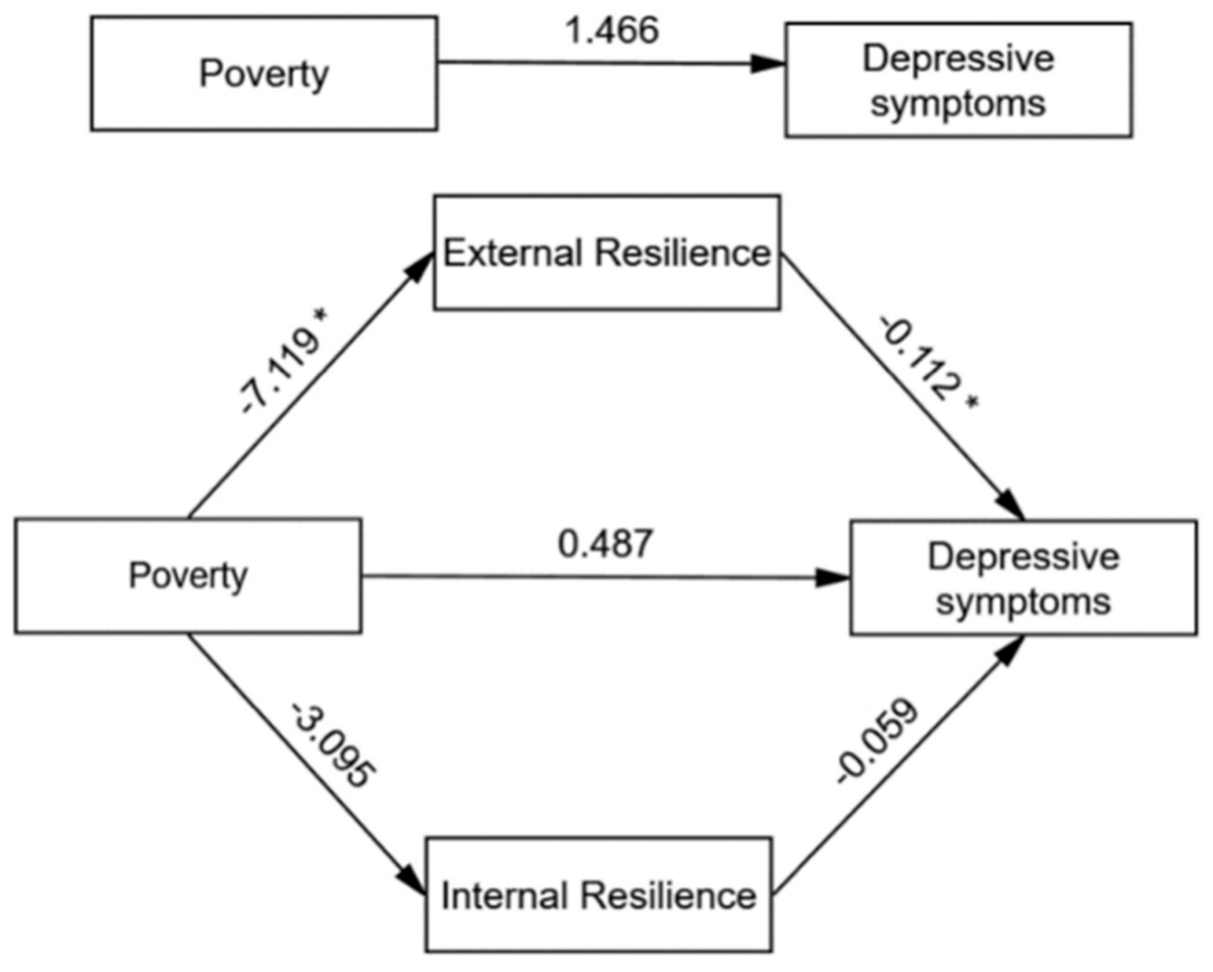
Model of the mediating role of resilience between poverty and depressive symptoms showing unstandardized path coefficients. Note:* Significant at 0.01 level.

**Fig. 2:**
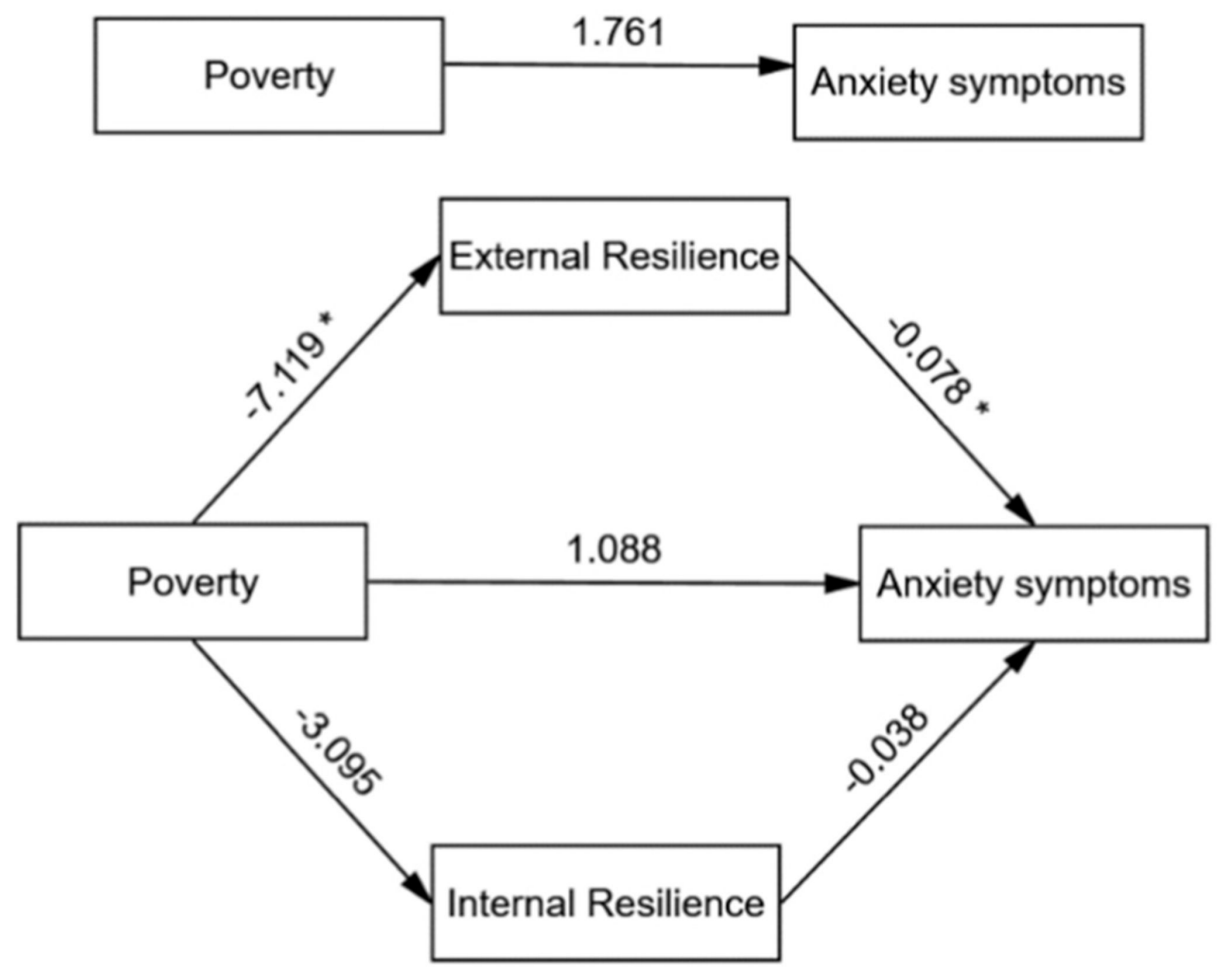
Model of the mediating role of resilience between poverty and anxiety symptoms showing unstandarized path coefficients. Note: * Significant at 0.01 level.

The results indicated that the total effect of poverty on depression was positive but not statistically significant (β = 1.466), and the direct effect of poverty on depression, after accounting for the mediators, also remained positive but non-significant (β = 0.487). However, the association between poverty and depression was significantly mediated through indirect effect via external resilience (β = 0.797, Boot SE 0.384; 95 % Boot CI 0.181, 1.677), indicating that poverty may increase depression by reducing external resilience. The indirect effect via internal resilience was also positive (β = 0.183, Boot SE 0.206) but not statistically significant (95 % Boot CI −0.141, 0.673). The total indirect effect through both mediators combined was significant (β = 0.979, Boot SE 0.415; 95 % Boot CI 0.234, 1.869), suggesting that resilience, particularly external resilience, plays a key mediating role in the relationship between poverty and depression (see Table 3). The parallel mediation model predicting depressive symptoms from poverty and resilience (both internal and external) explained 10.49 % of the variance in depressive symptoms [R^2^ = 0.1049; F (4, 485) = 14.21, *p* < 0.001]. The findings also indicated that gender significantly predicted depressive symptoms (β = 1.624, 95 % CI [0.834, 2.415]), with females reporting higher depression scores than males. Gender did not significantly predict either mediator: external resilience (β = −0.276, 95 % CI [−1.795, 1.243]) or internal resilience (β = −0.897, 95 % CI [−1.901, 0.108]), suggesting no meaningful gender differences for the role of resilience processes within the sample of adolescents. This likely reflects an attenuation of the already weak gender effect on the mediators—particularly on internal resilience, as suggested by the bivariate correlation (*r* = − 0.091 in Table 2)—once other variables were accounted for in the full model. The results from the parallel mediation models are presented in Fig. 1 and Fig. 2.

**Fig. 3:**
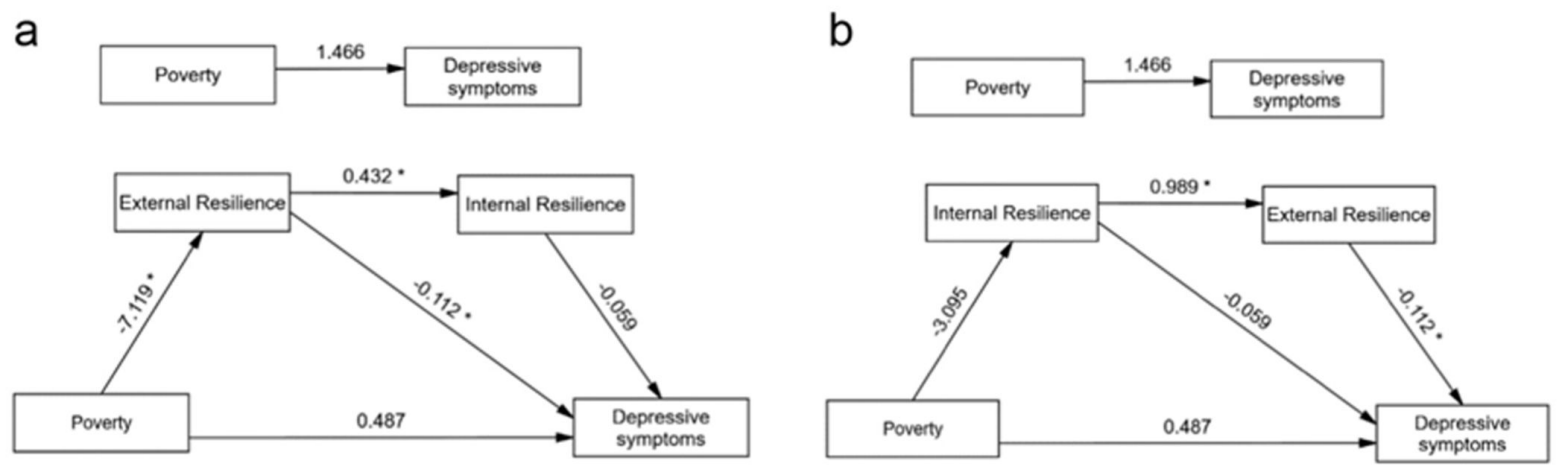
a Serial model of the mediating role of resilience between poverty and depressive symptoms showing unstandardized path coefficients. Note: * Significant at 0.01 level. b Serial model of the mediating role of resilience between poverty and depressive symptoms showing unstandardized path coefficients. Note: * Significant at 0.01 level.

The post-hoc exploratory serial mediation analysis examined whether the effect of poverty on depression symptoms operates through a causal chain linking external resilience and internal resilience, with results showing poverty significantly predicted lower external resilience (β = −7.119, *p* = 0.007), which in turn predicted higher internal resilience (β = 0.432, p < 0.001) (Fig. 3a). External resilience significantly predicted lower depression (β = −0.112, p < 0.001), whereas internal resilience did not (β = −0.059, *p* = 0.209). The direct effect of poverty on depression remained nonsignificant (β = 0.487, *p* = 0.725). An alternative pathway—poverty → internal resilience → external resilience → depression—was also explored (Fig. 3b). While poverty was associated with lower internal resilience, this effect was not statistically significant (β = −3.095, *p* = 0.078), although internal resilience was significantly associated with higher external resilience (β = 0.989, p < 0.001). These findings suggest a partial indirect effect primarily via external resilience in the association between poverty and depressive symptoms, without support for the full serial mediation pathway. The serial mediation model predicting depressive symptoms from poverty through external and internal resilience (considering either of the alternative pathways) explained 10.49 % of the variance in depressive symptoms [R^2^ = 0.1049; F (4, 485) = 14.21, p < 0.001].

**Fig. 4:**
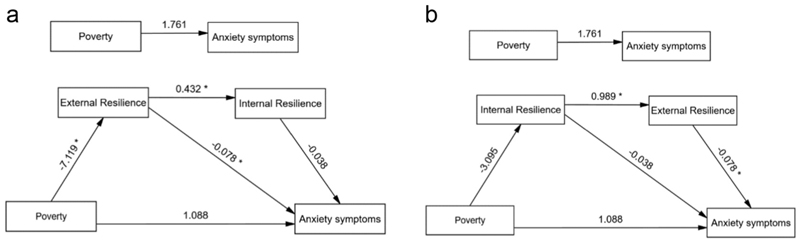
a Serial model of the mediating role of resilience between poverty and anxiety symptoms showing unstandardized path coefficients. Note: * Significant at 0.01 level. b Serial model of the mediating role of resilience between poverty and anxiety symptoms showing unstandardized path coefficients. Note: * Significant at 0.01 level.

Similar findings were evident in the parallel mediation model with the anxiety symptoms as the outcome (see Table 3). The results indicated that the total effect of poverty on anxiety was positive but not statistically significant (β = 1.761), and the direct effect of poverty on anxiety, after accounting for the mediators, also remained positive but non-significant (β = 1.088). However, the association between poverty and depression was significantly mediated through an indirect effect via external resilience (β = 0.557, Boot SE 0.294; 95 % Boot CI 0.101, 1.237), indicating that poverty may increase anxiety by reducing external resilience. The indirect effect via internal resilience was also positive (β = 0.117, Boot SE 0.165) but not statistically significant (95 % Boot CI −0.160, 0.498). The total indirect effect through both mediators combined was significant (β = 0.674, Boot SE 0.309; 95 % Boot CI 0.150, 1.355), suggesting that resilience, particularly external resilience, plays a key mediating role in the relationship between poverty and anxiety. The model predicting anxiety symptoms from poverty and resilience (both internal and external) explained 9.35 % of the variance in anxiety symptoms [R^2^ = 0.0935; F (4, 485) =12.51, *p* < 0.001]. Gender significantly predicted anxiety (β = 1.521, 95 % CI [0.852, 2.189]), with females reporting higher anxiety scores than males. Gender did not significantly predict either mediator: CYRMR (β = −0.276, 95 % CI [−1.795, 1.243]) or RRM (β = − 0.897, 95 % CI [− 1.901, 0.108]), suggesting no meaningful gender differences for resilience processes within this sample of adolescents. This suggests that the gender’s association with the mediators weakened when adjusted for additional variables in the full model. The post-hoc exploratory serial mediation analysis examined whether the effect of poverty on anxiety symptoms operates through a causal chain linking external resilience and internal resilience, with results showing poverty significantly predicted lower external resilience (β = −7.119, *p* = 0.007), which in turn predicted higher internal resilience (β = 0.432, p < 0.001) (Fig. 4a). External resilience significantly predicted lower anxiety (β = −0.078, *p* = 0.003), whereas internal resilience did not (β = −0.038, *p* = 0.342). The direct effect of poverty on anxiety remained nonsignificant (β = 1.088, *p* = 0.353). An alternative pathway—poverty → internal resilience → external resilience → anxiety—was also explored (Fig. 4b). While poverty was associated with lower internal resilience, this effect was not statistically significant (β = −3.095, *p* = 0.078), although internal resilience was significantly associated with higher external resilience (β = 0.989, p < 0.001). These findings suggest a partial indirect effect primarily via external resilience in the association between poverty and anxiety symptoms, without support for the full serial mediation pathway. The serial mediation model predicting depressive symptoms from poverty through external and resilience (considering either of the alternative pathways) explained 10.49 % of the variance in depressive symptoms [R^2^ = 0.0935; F (4, 485) =12.51, p < 0.001].

## 4 Discussion

The major finding of the current investigation shows the mediating effects of resilience in the association between poverty and symptoms of depression and anxiety among Nepali adolescents. Poverty’s effect on both depression and anxiety symptoms is mediated significantly through external resilience (i.e., indirect-only mediation), with no significant direct effect remaining after accounting for the mediators (internal and external resilience). The lack of a significant total and direct association between poverty and adolescent depression/anxiety symptoms is likely attributable to the study participants’ selection criteria, resulting in a homogeneously poor sample by design. This restricted variance in poverty attenuates the observable direct association, yet it strengthens the rationale for mediation analysis by highlighting the importance of underlying mechanisms. In deprived settings, where socioeconomic conditions are relatively uniform, individual variation in mental health outcomes, including depression and anxiety, may be more meaningfully explained by psychosocial factors such as resilience than by poverty itself.

This supports the findings that resilience processes, particularly external resilience, play a significant mediating role in the relationship between poverty and depression and anxiety, even within homogenously poor populations. Significant indirect effects were observed for both depressive (β = 0.797) and anxiety symptoms (β = 0.557) in the parallel mediation models, suggesting that resilience may help explain how structural stressors such as poverty affect psychological well-being. In short, higher poverty is associated with lower external resilience, which in turn is linked to greater depressive and anxiety symptoms. Internal resilience did not significantly mediate either association. While estimates pointed to a sizable portion of the association being transmitted through the external resilience pathway—54.33 % for depressive symptoms and 31.61 % for anxiety symptoms—these figures should be interpreted with caution, particularly given the absence of a statistically significant total effect. However, the presence and direction of the significant indirect effects of external resilience underscore the importance of psychosocial mechanisms in shaping adolescent mental health in deprived contexts. In the serial mediation model, the only significant indirect effects were again through external resilience alone, while no sequential mediation pathways involving both mediators were significant. These findings indicate that resilience may function as an independent factor with no sequential process and external resilience as a key mechanism linking poverty to adolescent mental health, particularly in homogenously poor contexts where individual internal protective factors may be less developed or less impactful. For anxiety and depressive symptoms as outcomes, the mediation models significantly explained 9.35 % and 10.49 % of the variances, respectively. Moreover, our mediation models suggest that poverty exerts a comparatively stronger influence on resilience (external over internal) than outcome variables (depression and anxiety) included in the models. This indicates that poverty-related adversity may play a central role in shaping resilience among adolescents. From an intervention perspective, this highlights the importance of addressing structural and socioeconomic disadvantages alongside individual- and community-level strategies to strengthen resilience and reduce risks of depression and anxiety.

A similar result was obtained by Kim et al. (2016) among 13–17-year-old low- and middle-income adolescents, who found that poverty exposure from birth to early adolescence predicted higher use of disengagement coping (i.e., reduced element of internal resilience), which in turn was associated with increased internalizing symptoms. Nurius et al. (2020) reported that family bonding and engagement in school (i.e., external resilience resources), among adolescent students in Washington State, helped protect against the harmful impact of growing up in poverty and thereby, ultimately had a positive influence on the mental well-being of adolescents and youth. School engagement, measured as feeling connected with school, safe at school, and having attachment relationships with peers and teachers, has been shown to yield behavioral health advantages both during adolescence (Bond et al., 2007) and later in adulthood (Steiner et al., 2021). In contrast, parental rejection worsens the relationship between parental stress and adolescents’ mental health (Sofrona and Giannakopoulos, 2024), implying that parental acceptance might serve as a potential external resilience factor. This may be particularly important in Nepal where the traditional societal norms often support parents as the primary source of social support to validate their children’s feelings and strengths. Youth reporting less closeness with their parents have been found to have reduced activation in the dorsolateral prefrontal cortex, which may ultimately lead to diminished emotion regulation and inhibitory control (Morningstar et al., 2019). This reduced neural regulation, potentially stemming from weaker parental bonds, could contribute to lower protective factors among adolescents.

While the role of external protective factors—particularly social support—is well-established in global literature (Robles, 2021; Perry et al., 2021; Chen et al., 2017; Gunnar, 2017), this study adds a culturally grounded perspective from Nepal that helps refine and contextualize prevailing resilience models. In many Western-centric frameworks, internal resilience (e.g., self-regulation, self-efficacy, optimism, perseverance) is often emphasized as a mechanism of mental health promotion and protection (Eadeh et al., 2021; Jefferies et al., 2022). However, our findings suggest that in the Nepali context, which is strongly shaped by collectivist cultural values, influenced by familial norms and communal living, adolescents tend to rely more on social ways of coping, such as seeking family and social support and inter-connectedness, with less emphasis on independent, internal strategies. In this context, external resilience played a more prominent mediating role among young adolescents who are living in homogenously deprived conditions. This indicates that access to supportive relationships and community-based resources may not just be helpful, but essential in coping with adversity, such as that brought about by poverty among adolescents. These findings point to the need for tailoring culturally adapted interventions that prioritize strengthening external support systems for young adolescents living in deprivation in Nepal, and possibly other collectivist cultures. The findings of this study could help shape the design of school-based or community-level mental health programs in Nepal that invest in peer support networks, family engagement, and local mentorship—strategies that fit well with the culture and available resources.

External resilience played a stronger mediating role than internal resilience in the study might also be due to developmental factors in adolescence—a phase marked by neuroplasticity, social reorientation, and the ongoing maturation of executive functions (Masten and Barnes, 2018; Casey et al., 2005; Baker et al., 2025; Irani et al., 2024). During this period, social support tends to buffer stress more effectively than internal traits like ruggedness, which typically develop as the brain matures later through experience and exploration (Romer et al., 2017). In this study, where participants lived in homogenously deprived settings, internal protective factors such as meaning-making in life or a strong sense of purpose were likely less developed and less impactful, also considering the young age (13–15 years). Prior research in Nepal confirms that family-based social support significantly contributes to adolescent self-esteem and well-being (Poudel et al., 2020). Supportive relationships—within families, peer groups, and communities—are widely recognized as external protective factors (Chen et al., 2017; Chen et al., 2011; Li et al., 2019; Evans and Kim, 2013; Laible et al., 2000) that plays a key role in shaping resilience across the lifespan (Chiang et al., 2018; Luthar et al., 2015; Masten, 2014), often outweighing individual traits like personality or cognitive skills in fostering adaptive capacity. Ecological and transactional models (Bronfenbrenner, 1977; Cicchetti and Lynch, 1993) highlight how nested environmental contexts (e.g., culture, neighborhood, family), as sources of external resilience, shape child development. These frameworks have provided foundational concepts for resilience research, particularly in understanding adaptation amid risks like family poverty (Baldwin et al., 1993). Moreover, the ecological model of development suggests that without supportive external structures or resources, the efficacy of internal traits could be undermined, supporting the finding that rugged qualities of an adolescent might not be sufficient without an adequate protective external environment. For instance, a child’s problem-solving ability may not be effective in the absence of a caregiver/teacher’s support, academic opportunities, or a safe family or school environment.

These frameworks have provided foundational concepts for resilience research, particularly in understanding adaptation amid risks like family poverty (Baldwin et al., 1993). Moreover, the ecological model of development suggests that without supportive external structures or resources, the efficacy of internal traits could be undermined, supporting the finding of this study that rugged qualities of an adolescent might not be sufficient without an adequate protective external environment. For instance, a child’s problem-solving ability may not be effective in the absence of a caregiver/teacher’s support, academic opportunities, or a safe family or school environment. This study supports the concept of co-regulation in adolescents and underscores the importance of including caregiver interventions alongside adolescent-focused interventions for the prevention of depression and anxiety, particularly among Nepalese adolescents from a collectivist family culture, consistent with the ALIVE pilot trial modality (Jordans et al., 2025). The findings further highlight the significance of resilience—especially external resilience—as a key construct in adolescent mental health in this context. By identifying levels of resilience within the study population, the results emphasize how resilience can inform the targeting of preventive interventions. Additionally, the study demonstrates the value of examining mechanisms in low-resource contexts, indicating that external support systems are critical mechanisms that could be leveraged to enhance the effectiveness of future interventions. Poverty was negatively correlated with resilience in this study. Poverty is one of the family-level stressors that can threaten the connectedness within a family (i.e., primary source of comfort and support during times of stress), reducing the likelihood of resilient outcomes in children and adolescents (Lynch and Cicchetti, 1998). Poverty can also be considered developmentally challenging because of its effects on adolescents: undermining emotional security, dislodging adaptability, and impeding the use of existing resources or navigating new ones (Bradley, 2007). While gender was examined due to reported higher mental health risks among females, the lack of a significant association with resilience mediators might reflect Nepal’s collectivist culture, where certain forms of social support such as that from family members or peers or school teachers may be equally accessible or valued across genders, or it could point to specific developmental patterns in the studied age group of 13–15 years adolescents. The present study also showed that there was a high correlation (*r* = 0.656) between internal resilience and external resilience among adolescents. This finding aligns with previous studies, which suggested that an individual may enhance feelings of internal resilience by drawing upon the sources of external resilience, which strengthen a sense of agency by accessing sources of external resilience in the future; thus, internal and external resilience are strongly related (Tomko et al., 2022).

While previous studies have consistently shown that poverty negatively impacts adolescent mental well-being, addressing poverty itself requires large-scale, multi-sectoral interventions that take considerable time and resources. Literature has also suggested efforts to focus on understanding and targeting the underlying mechanisms through which poverty affects mental health (Ridley et al., 2020). The mediational findings in the present study identify resilience as a mechanism between poverty and adolescent depression and anxiety, and thereby support the socio-ecological and stress buffering framework that external protective factors, such as interaction with the social and cultural environment (the active ingredients of external resilience), can buffer the psychological impact of material deprivation in low-resource setting. Therefore, policy efforts and targeted interventions should be integrated within multi-layered approaches—poverty reduction programs, for instance, can be designed to simultaneously strengthen internal and external protective resilience factors by fostering supportive environments and empowering adolescents. The effectiveness of specific protective factors may depend on the nature of the adversity faced; for example, perseverance and problem-solving might be more relevant to challenges like academic stress, peer conflict, experiences of bullying, or family disruption than to structural stressors such as poverty, which adolescents may have limited agency to address while still living with parents. This highlights the importance of considering the fit between protective factors and types of adversity—a direction that warrants greater attention in future resilience research.

Consistent with prior evidence, mostly from high-income countries, this study is the first of its kind in Nepal to empirically test the role of resilience in the relationship between poverty and adolescent depressive and anxiety symptoms. The findings of the mediation analyses suggest that for depression and anxiety prevention studies, such as the ALIVE pilot trial (Lund et al., 2023), the underlying mechanism of resilience for the interventions’ effects should be temporally tested in line with both internal and external protective factors. Furthermore, the prevention and promotion interventions could also focus on promoting protective factors of resilience, both external and internal, as they are often correlated and may lay the foundation for more rugged, adaptive qualities in adulthood, ultimately enhancing individuals’ capacity to face adversities and stressors across the lifespan. This opens two lines of enquiry for future studies – first, the studies should focus on resilience-building interventions/programs (e.g., life skills training such as negotiation involving communication and social interaction, social support structures, to strengthen the external protective factors) that may help reduce the mental health burden among adolescents living in stressors, but not limited to poverty. Second, while internal resilience factors such as perseverance and pride in achievement are commonly associated with positive outcomes, their relevance may vary depending on the type of adversity—qualities like problem-solving may interface more directly with the challenges posed by poverty, whereas other internal factors may have limited utility in such contexts. This underscores the need for future research to unpack the relative and interacting contributions of individual protective factors within tools like the RRM and CYRM-R, particularly in culturally specific settings, to better inform targeted “resilience-building” interventions for youth facing structural hardship.

There are a few limitations of this study. First, the use of self-report measures and cross-sectional data might lead to biased estimates. Due to the cross-sectional design of the study, the mediation analysis lacks a temporal dimension, meaning the observed associations may be due to reverse causality. Second, the sample included homogeneously poor adolescents, which may explain the lack of significant association between poverty and adolescent depression and/or anxiety. However, this emphasizes the central role of resilience-building programs to promote mental well-being, particularly in contexts where poverty is widespread. Third, there was a high correlation between the two mediators (i.e., RRM and CYRMR). However, diagnostic checks indicated that multi-collinearity was not serious enough to compromise the integrity of the mediation analysis. The parallel mediation followed by additional exploratory serial mediation analyses conclusively help determine the mediational effects in parallel and serial mediation models (and proportion of indirect effect over total effect), and showed some distinct features that the external resilience impact outweighed the internal resilience among adolescents in this study. Another limitation is that we did not adjust for grade level, age, or ethnicity in the mediation models. Grade was used only to select participants within the target age group, age variability was minimal (13–15 years), and ethnicity showed no significant associations with depression or anxiety in bivariate analyses. Including these variables would also have required multiple dummy codes, which was not feasible given the modest sample size and model complexity. Lastly, the sample was recruited exclusively from grades 6 to 8, encompassing 13–15-year-old students, which may not represent adolescents of older ages.

## 5 Conclusion

External resilience consistently emerged as a significant mediator across both parallel and serial mediation models, explaining the relationship between poverty and depression and anxiety symptoms in Nepali adolescents, even in the context of relatively homogenous socioeconomic adversity. Although the total association of poverty with depression and anxiety was not statistically significant—likely due to the homogeneity of the sample—external resilience accounted for 54.33 % of the association of poverty with depression and 31.61 % with anxiety, contributing to total indirect effects of 66.79 % and 38.25 %, respectively. This means external protective factors such as family and social support, interconnectedness, and social ways of coping might play a central role in buffering against the impact of structural stressors like poverty, particularly among homogeneously poor populations. The same mediation was not found for internal resilience, possibly because of Nepal’s strong and unique collectivist culture with profound familial and communal norms emphasizing external support systems during adolescence. Internal protective factors, such as developing the strong sense of purpose or meaning-making and perseverance may be less relevant and impactful to cope against structural stressors such as poverty in this context. Overall, resilience, particularly external resilience, plays a significant mediating role, suggesting that it is a promising mechanism for more targeted mental health interventions in young populations, particularly adolescents. Future studies should use longitudinal data to confirm causality and test the effectiveness of mental health promotion and prevention programs among adolescents with resilience, particularly external resilience, as a mechanism.

Supplementary data to this article can be found online at https://doi.org/10.1016/j.jad.2025.120589.

## Supplementary Material

Table S1

## Figures and Tables

**Table 1:** Participant characteristics (*n* = 490).

Characteristics and categories	Frequency (%) or mean (SD)
Age	13.85 (0.73)^a^
Gender	
Male	232 (47.3)
Female	258 (52.7)
Grade	
Sixth	109 (22.2)
Seventh	210 (42.9)
Eighth	171 (34.9)
Ethnicity	
Janajati	247 (50.4)
Brahmin or Chhetri	134 (27.3)
Dalit	51 (10.4)
Madhesi	37 (7.6)
Muslim	20 (4.1)
Don’t know/Prefer not to say	1 (0.2)

a Mean (SD).

**Table 2:** Mean, SD, and bivariate correlations between variables (n = 490).

Variables	Mean	SD	Poverty	PHQA	GAD7	CYRMR	RRM	Gender
Poverty	0.59	0.15	–					
PHQA	8.46	4.61	0.072	–				
GAD7	6.13	3.88	0.095*	0.779**	–			
CYRMR	66.88	8.51	− 0.124**	− 0.261**	− 0.219**	–		
RRM	37.35	5.62	− 0.092*	− 0.225**	− 0.189**	0.656**	–	
Gender^a^	–	–	0.141**	0.192**	0.212**	− 0.033	− 0.091*	–

* Correlation significant at 0.05 level.

** Correlation significant at 0.001 level.

a Correlation between gender (coded 0 = male, 1 = female) and continuous variables represents the point-biserial correlation.

**Table 3:** Bootstrapping indirect effects and 95 % confidence intervals (CI) for the mediational analyses in the relationship between poverty and depressive and anxiety symptoms.

Path description/models	β coefficient (unstandardized effect)	SE	95 % CI	*p*-Value	Proportion of mediating effect
Parallel Mediation Models					
Poverty → CYRMR	– 7.119	2.647	–12.321, –1.917	0.007*	–
Poverty → RRM	– 3.095	1.750	–6.535, 0.344	0.078	–
CYRMR → depressive symptoms	– 0.112	0.031	–0.173, –0.051	<0.001*	–
RRM → depressive symptoms	– 0.059	0.047	– 0.151, 0.033	0.209	–
Poverty → CYRMR → depressive symptoms (indirect effect)	0.797	0.384	0.181, 1.677	–	54.33 %
Poverty → RRM → depressive symptoms (indirect effect)	0.183	0.206	– 0.141, 0.673	–	12.45 %
Poverty → Both CYRMR and RRM → depressive symptoms (indirect effect)	0.979	0.415	0.234, 1.869	–	66.79 %
CYRMR → anxiety symptoms	– 0.078	0.026	–0.130, 0.027	0.003*	–
RRM → anxiety symptoms	– 0.038	0.040	– 0.116, 0.040	0.342	–
Poverty → CYRMR → anxiety symptoms (indirect effect)	0.557	0.294	0.101, 1.237	–	31.61 %
Poverty → RRM → anxiety symptoms (indirect effect)	0.117	0.165	–0.160, 0.498	–	6.63 %
Poverty → Both CYRMR and RRM → anxiety symptoms (indirect effect)	0.674	0.309	0.150, 1.355	–	38.25 %
Poverty → depressive symptoms (direct effect)	0.487	1.382	– 2.228, 3.202	0.725	–
Poverty → anxiety symptoms (direct effect)	1.088	1.169	–1.209, 3.384	0.353	–
Poverty → depressive symptoms (total effect)	1.466	1.419	–1.321, 4.254	0.302	–
Poverty → anxiety symptoms (total effect)	1.761	1.186	–0.568, 4.091	0.138	–
Serial Mediation Models					
Poverty → CYRMR	– 7.119	2.647	–12.320, –1.917	0.007*	–
CYRMR → RRM	0.432	0.023	0.388, 0.477	<0.001*	–
Poverty → RRM	– 3.095	1.750	–6.535, 0.344	0.078	–
RRM → CYRMR	0.989	0.052	0.887, 1.091	<0.001*	–
CYRMR → depressive symptoms	– 0.112	0.031	–0.173, –0.051	<0.001*	–
RRM → depressive symptoms	– 0.059	0.047	– 0.151, 0.033	0.209	–
Poverty → CYRMR → RRM → depressive symptoms (indirect effect)	0.181	0.186	– 0.119, 0.618	–	12.35 %
Poverty → RRM → CYRMR → depressive symptoms (indirect effect)	0.342	0.221	–0.024, 0.847	–	23.33 %
Poverty → CYRMR → depressive symptoms (indirect effect only through external resilience)	0.797	0.385	0.164, 1.665	–	54.37 %
Poverty → RRM → depressive symptoms (indirect effect only through internal resilience)	0.183	0.207	–0.136, 0.689	–	12.48 %
CYRMR → anxiety symptoms	– 0.078	0.026	–0.130, –0.027	0.003*	–
RRM → anxiety symptoms	– 0.038	0.040	– 0.116, 0.040	0.342	–
Poverty → CYRMR → RRM → anxiety symptoms (indirect effect)	0.116	0.143	– 0.141, 0.437	–	6.59 %
Poverty → RRM → CYRMR → anxiety symptoms (indirect effect)	0.239	0.164	–0.033, 0.623	–	13.57 %
Poverty → CYRMR → anxiety symptoms (indirect effect only through external resilience)	0.557	0.297	0.095, 1.229	–	31.63 %
Poverty → RRM → anxiety symptoms (indirect effect only through internal resilience)	0.117	0.169	–0.162, 0.527	–	6.64 %
Poverty → depressive symptoms (direct effect)	0.487	1.382	– 2.228, 3.202	0.725	–
Poverty → anxiety symptoms (direct effect)	1.088	1.169	–1.209, 3.384	0.353	–
Poverty → depressive symptoms (total effect)	1.466	1.419	–1.321, 4.254	0.302	–
Poverty → anxiety symptoms (total effect)	1.761	1.186	–0.568, 4.091	0.138	–

Note: All models were adjusted for gender; * Significant at 0.01 level; both CYRMR and RRM indicate the total indirect effect when both mediators are included in the parallel mediation model, representing the sum of their specific indirect effects.

## Data Availability

All required data are available within the manuscript. The data that support the findings of this study are part of a larger research project and are not publicly available. However, they may be made available from the corresponding author upon reasonable request.

## References

[R1] Baker AE, Galván A, Fuligni AJ (2025). The connecting brain in context: how adolescent plasticity supports learning and development. Dev Cogn Neurosci.

[R2] Baldwin AL, Baldwin CP, Kasser T, Zax M, Sameroff A, Seifer R (1993). Contextual risk and resiliency during late adolescence. Dev Psychopathol.

[R3] Betancourt TS, Khan KT (2008). The mental health of children affected by armed conflict: protective processes and pathways to resilience. Int Rev Psychiatry.

[R4] Blakemore S, Burnett S, Dahl RE (2010). The role of puberty in the developing adolescent brain. Hum Brain Mapp.

[R5] Bond L, Butler H, Thomas L, Carlin J, Glover S, Bowes G, Patton G (2007). Social and school connectedness in early secondary school as predictors of late teenage substance use, mental health, and academic outcomes. J Adolesc Health.

[R6] Bradley RH (2007). Parenting in the breach: how parents help children cope with developmentally challenging circumstances. Parenting.

[R7] Bronfenbrenner U (1977). Toward an experimental ecology of human development. Am Psychol.

[R8] Carvajal-Velez L, Requejo JH, Ahs JW, Servili C, Wasserman D, Kohrt BA (2023). Increasing data and understanding of adolescent mental health worldwide: UNICEF’s measurement of mental health among adolescents at the population level initiative. J Adolesc Health.

[R9] Casey BJ, Tottenham N, Liston C, Durston S (2005). Imaging the developing brain: what have we learned about cognitive development? Trends Cogn. Sci.

[R10] Chen E, Miller GE, Kobor MS, Cole SW (2011). Maternal warmth buffers the effects of low early-life socioeconomic status on pro-inflammatory signaling in adulthood. Mol Psychiatry.

[R11] Chen E, Brody GH, Miller GE (2017). Childhood close family relationships and health. Am Psychol.

[R12] Chiang JJ, Chen E, Miller GE (2018). Midlife self-reported social support as a buffer against premature mortality risks associated with child abuse. Nat Hum Behav.

[R13] Cicchetti D, Lynch M (1993). Toward an ecological/transactional model of community violence and child maltreatment: consequences for children’s development. Psychiatry.

[R14] Dangal MR, Bajracharya LS (2020). Students’ anxiety experiences during COVID-19 in Nepal. Kathmandu Univ Med J.

[R15] Díaz Y, Hessel P, Avendano M, Evans-Lacko S (2022). Multidimensional poverty and adolescent mental health: unpacking the relationship. Soc Sci Med (1982).

[R16] Eadeh HM, Breaux R, Nikolas MA (2021). A meta-analytic review of emotion regulation focused psychosocial interventions for adolescents. Clin Child Fam Psychol Rev.

[R17] Erskine HE, Moffitt TE, Copeland WE, Costello EJ, Ferrari AJ, Patton G, Degenhardt L, Vos T, Whiteford HA, Scott JG (2015). A heavy burden on young minds: the global burden of mental and substance use disorders in children and youth. Psychol Med.

[R18] Evans GW, Kim P (2013). Childhood poverty, chronic stress, self-regulation, and coping. Child Dev Perspect.

[R19] Fritz MS, MacKinnon DP (2007). Required sample size to detect the mediated effect. Psychol Sci.

[R20] Gautam P, Dahal M, Ghimire H, Chapagain S, Baral K, Acharya R, Neupane A (2021). Depression among adolescents of rural Nepal: a community-based study. Depress Res Treat.

[R21] Ghimire R, Adhikari Mishra T, Sharma P (2024). Stress, anxiety, and depression among adolescents of schools in Godawari Municipality, Lalitpur. Kathmandu Univ Med J.

[R22] Giri R, Khadka S, Chalise A, Swar K, Paudel S (2024). Depressive symptoms and its associated factors among secondary school adolescents of Birtamod Municipality, Jhapa, Nepal. PLoS Glob Public Health.

[R23] Gunnar MR (2017). Social buffering of stress in development: a career perspective. Perspect Psychol Sci.

[R24] Hayes AF (2009). Beyond Baron and Kenny: statistical mediation analysis in the new millennium. Commun Monogr.

[R25] Hielscher E, Blake J, Chang I, Crandon T, McGrath M, Scott JG (2023). Sense of purpose interventions for depression and anxiety in youth: a scoping review and cross-cultural youth consultation. J Affect Disord.

[R26] IBM Corp (2022). IBM SPSS Statistics for Windows (Version 290) [Computer software].

[R27] Irani F, Muotka J, Lyyra P, Parviainen T, Monto S (2024). Social influence in adolescence: behavioral and neural responses to peer and expert opinion. Soc Neurosci.

[R28] Jefferies P, McGarrigle L, Ungar M (2019). The CYRM-R: a Rasch-validated revision of the child and youth resilience measure. J EvidBased Soc Work.

[R29] Jefferies P, Vanstone R, Ungar M (2022). The rugged resilience measure: development and preliminary validation of a brief measure of personal resilience. Appl Res Qual Life.

[R30] Jordans M, Lund C, Garman E, Bonifaz MS, Avendano M, Bauer A, Yarrow P (2025). Combining self-regulation and poverty reduction to prevent depression and anxiety amongst adolescents experiencing multi-dimensional poverty in Colombia, Nepal and South Africa: Study protocol of a pilot 4-arm cluster Randomized Controlled Trial.

[R31] Karki A, Thapa B, Pradhan PMS, Basel P (2022). Depression, anxiety and stress among high school students: a cross-sectional study in an urban municipality of Kathmandu, Nepal. PLOS Glob Public Health.

[R32] Kieling C, Baker-Henningham H, Belfer M, Conti G, Ertem I, Omigbodun O, Rohde LA, Srinath S, Ulkuer N, Rahman A (2011). Child and adolescent mental health worldwide: evidence for action. Lancet.

[R33] Kim P, Neuendorf C, Bianco H, Evans GW (2016). Exposure to childhood poverty and mental health symptomatology in adolescence: a role of coping strategies. Stress Health.

[R34] Kroenke K, Spitzer RL, Williams JBW (2001). The PHQ-9: validity of a brief depression severity measure. J Gen Intern Med.

[R35] Laible DJ, Carlo G, Raffaelli M (2000). The differential relations of parent and peer attachment to adolescent adjustment. J Youth Adolesc.

[R36] Lane R, Taylor H, Ellis F, Rushworth I, Chiu K (2025). Resilience and its association with mental health among forcibly displaced populations: a systematic review and meta-analyses. J Affect Disord.

[R37] Li C, Wu Q, Liang Z (2019). Effect of poverty on mental health of children in rural China: the mediating role of social capital. Appl Res Qual Life.

[R38] Lin J, Guo W (2024). The research on risk factors for adolescents’ mental health. Behav Sci.

[R39] Liu S, Yu B, Xu C, Zhao M, Guo J (2022). Characteristics of collective resilience and its influencing factors from the perspective of psychological emotion: a case study of COVID-19 in China. Int J Environ Res Public Health.

[R40] Luitel NP, Rimal D, Eleftheriou G, Rose-Clarke K, Nayaju S, Gautam K, Pant SB, Devkota N, Rana S, Chaudhary JM, Gurung BS (2024). Translation, cultural adaptation and validation of Patient Health Questionnaire and generalized anxiety disorder among adolescents in Nepal. Child Adolesc Psychiatry Ment Health.

[R41] Lund C, Breen A, Flisher AJ, Kakuma R, Corrigall J, Joska JA, Swartz L, Patel V (2010). Poverty and common mental disorders in low and middle income countries: a systematic review. Soc Sci Med.

[R42] Lund C, Jordans MJD, Garman E, Araya R, Avendano M, Bauer A, Yarrow P (2023). Strengthening self-regulation and reducing poverty to prevent adolescent depression and anxiety: rationale, approach and methods of the ALIVE interdisciplinary research collaboration in Colombia, Nepal and South Africa. Epidemiol Psychiatr Sci.

[R43] Luthar SS, Crossman EJ, Small PJ, Lerner RM, Lamb ME (2015). Handbook of Child Psychology and Developmental Science.

[R44] Lynch M, Cicchetti D (1998). An ecological-transactional analysis of children and contexts: the longitudinal interplay among child maltreatment, community violence, and children’s symptomatology. Dev Psychopathol.

[R45] Masten AS (2014). Ordinary Magic: Resilience in Development.

[R46] Masten AS, Barnes AJ (2018). Resilience in children: developmental perspectives. Children.

[R47] Masten AS, Wright MO, Reich JW, Zautra AJ, Hall JS (2010). Handbook of Adult Resilience.

[R48] Mesman E, Vreeker A, Hillegers M (2021). Resilience and mental health in children and adolescents: an update of the recent literature and future directions. Curr Opin Psychiatry.

[R49] Michelson D, Hodgson E, Bernstein A, Chorpita BF, Patel V (2022). Problem solving as an active ingredient in indicated prevention and treatment of youth depression and anxiety: an integrative review. J Adolesc Health.

[R50] Morningstar M, Grannis C, Mattson WI, Nelson EE (2019). Associations between adolescents’ social re-orientation toward peers over caregivers and neural response to teenage faces. Front Behav Neurosci.

[R51] Mudunna C, Weerasinghe M, Tran T, Antoniades J, Romero L, Chandradasa M, Fisher J (2025). Nature, prevalence and determinants of mental health problems experienced by adolescents in South Asia: a systematic review. Lancet Reg Health Southeast Asia.

[R52] Najman JM, Hayatbakhsh MR, Clavarino A, Bor W, O’Callaghan MJ, Williams GM (2010). Family poverty over the early life course and recurrent adolescent and young adult anxiety and depression: a longitudinal study. Am J Public Health.

[R53] Nepal Health Research Council (2021). Report of National Mental Health Survey 2020.

[R54] Nurius P, LaValley K, Kim MH (2020). Victimization, poverty, and resilience resources: stress process considerations for adolescent mental health. Sch Ment Health.

[R55] Ojha SP, Ma J, Chapagain M, Tulachan P (2013). Emotional and behavioural problems among sheltered homeless children. J Nepal Med Assoc.

[R56] Pangallo A, Zibarras L, Lewis R, Flaxman P (2015). Resilience through the lens of interactionism: a systematic review. Psychol Assess.

[R57] Patel V, Kleinman A (2003). Poverty and common mental disorders in developing countries. Bull World Health Organ.

[R58] Patel V, Araya R, de Lima M, Ludermir A, Todd C (1999). Women, poverty and common mental disorders in four restructuring societies. Soc Sci Med.

[R59] Patton GC, Tollit MM, Romaniuk H, Spence SH, Sheffield J, Sawyer MG (2011). A prospective study of the effects of optimism on adolescent health risks. Pediatrics.

[R60] Paudel S, Gautam H, Adhikari C, Yadav DK (2020). Depression, anxiety and stress among the undergraduate students of Pokhara Metropolitan, Nepal. J Nepal Health Res Counc.

[R61] Perry N, Johnson A, Hostinar C, Gunnar M (2021). Parental emotional support and social buffering in previously institutionalized and typically developing children and adolescents. Dev Psychobiol.

[R62] Pieters R (2017). Meaningful mediation analysis: plausible causal inference and informative communication. J Consum Res.

[R63] Poudel A, Gurung B, Khanal GP (2020). Perceived social support and psychological wellbeing among Nepalese adolescents: the mediating role of self-esteem. BMC Psychol.

[R64] Preacher KJ, Hayes AF (2004). SPSS and SAS procedures for estimating indirect effects in simple mediation models. Behav Res Methods.

[R65] Preacher KJ, Hayes AF (2008). Asymptotic and resampling strategies for assessing and comparing indirect effects in multiple mediator models. Behav Res Methods.

[R66] Ridley M, Rao G, Schilbach F, Patel V (2020). Poverty, depression, and anxiety: causal evidence and mechanisms. Science.

[R67] Robles TF (2021). Annual research review: social relationships and the immune system during development. J Child Psychol Psychiatry.

[R68] Romer D, Reyna VF, Satterthwaite TD (2017). Beyond stereotypes of adolescent risk taking: placing the adolescent brain in developmental context. Dev Cogn Neurosci.

[R69] Ruan QN, Chen C, Jiang D-G, Yan W-J, Lin Z (2022). A network analysis of social problem-solving and anxiety/depression in adolescents. Front Psych.

[R70] Sharma A, Kar N (2019). Posttraumatic stress, depression, and coping following the 2015 Nepal earthquake: a study on adolescents. Disaster Med Public Health Preparedness.

[R71] Shawon MSR, Hossain FB, Hasan M, Rahman MR (2024). Gender differences in the prevalence of anxiety and depression and care seeking for mental health problems in Nepal: analysis of nationally representative survey data. Glob Ment Health.

[R72] Shrout PE, Bolger N (2002). Mediation in experimental and nonexperimental studies: new procedures and recommendations. Psychol Methods.

[R73] Silwal S, Dybdahl R, Chudal R, Sourander A, Lien L (2018). Psychiatric symptoms experienced by adolescents in Nepal following the 2015 earthquakes. J Affect Disord.

[R74] Singh R, Chua KC, Pant SB, Paudel R, Gautam K, Luitel NP, Lund C (2025). Translation and adaptation of the Child and Youth Resilience Measure–Revised and Rugged Resilience Measure: A mixed-method study among adolescents in Nepal. Manuscript submitted for publication.

[R75] Sofrona E, Giannakopoulos G (2024). The impact of parental depressive, anxiety, and stress symptoms on adolescents’ mental health and quality of life: the moderating role of parental rejection. Children.

[R76] Sousa CA, Haj-Yahia MM, Feldman G, Lee J (2013). Individual and collective dimensions of resilience within political violence. Trauma Violence Abuse.

[R77] Spitzer RL, Kroenke K, Williams JBW, Löwe B (2006). A brief measure for assessing generalized anxiety disorder: the GAD-7. Arch Intern Med.

[R78] Steel Z, Chey T, Silove D, Marnane C, Bryant RA, van Ommeren M (2009). Association of torture and other potentially traumatic events with mental health outcomes among populations exposed to mass conflict and displacement: a systematic review and meta-analysis. JAMA.

[R79] Steiner RJ, Sheremenko G, Lesesne C, Dittus PJ, Sieving RE, Ethier KA (2021). Adolescent connectedness and adult health outcomes. Pediatrics.

[R80] Teare MD, Dimairo M, Shephard N, Hayman A, Whitehead A, Walters SJ (2014). Sample size requirements to estimate key design parameters from external pilot randomised controlled trials: a simulation study. Trials.

[R81] Thapar A, Pine DS, Leckman JF, Scott S, Snowling MJ, Taylor EA (2017). Rutter’s Child and Adolescent Psychiatry.

[R82] Tomko C, Nestadt DF, Weicker NP, Rudzinski K, Underwood C, Kaufman MR, Sherman SG (2022). External resilience in the context of drug use and socio-structural vulnerabilities: a qualitative exploration among women who use drugs and sell sex in Baltimore, Maryland. Harm Reduct J.

[R83] Ungar M (2011). The social ecology of resilience: addressing contextual and cultural ambiguity of a nascent construct. Am J Orthopsychiatry.

[R84] Ungar M, Theron L, Murphy K, Jefferies P (2021). Researching multisystemic resilience: a sample methodology. Front Psychol.

[R85] UNICEF (2021). The state of the world’s children 2021: On my mind – Promoting, protecting and caring for children’s mental health.

[R86] United Nations Development Programme (2024). Multidimensional Poverty Index 2024: Nepal.

[R87] Van Doren N, Tharp JA, Johnson SL, Staudenmaier PJ, Anderson C, Freeman MA (2019). Perseverance of effort is related to lower depressive symptoms via authentic pride and perceived power. Pers Individ Differ.

[R88] World Health Organization (2018). Disease, injury and causes of death regional estimates, 2004–2008.

[R89] World Health Organization (2024). Mental health of adolescents. [Fact sheet].

[R90] Zhao X, Lynch JG, Chen Q (2010). Reconsidering Baron and Kenny: myths and truths about mediation analysis. J Consum Res.

[R91] Zimmerman MA, Brenner AB, Reich JW, Zautra AJ, Hall JS (2010). Handbook of Adult Resilience.

