# Supplementary material for: Relationship between poverty and symptoms of depression and anxiety among adolescents in Nepal: Examining the mediating effect of external and internal resilience
Source: J Affect Disord. Author manuscript; Available in PMC 2025 Nov 23. (PMC7618394; doi:10.1016/j.jad.2025.120589)
Supplement: Table S1 [file EMS210910-supplement-Table_S1.docx]

*Table S1. Mean, SD, and bivariate correlations between poverty and categories of depression/anxiety symptoms (n=490)*

| Variables | N | Mean | SD | Correlation Coefficient | 95% CI |
| --- | --- | --- | --- | --- | --- |
| No/Minimal Depression | 108 | 2.96 | 1.06 | 0.099 | -0.091, 0.283 |
| Mild Depression | 207 | 7.09 | 1.41 | -0.139* | -0.270, -0.002 |
| Moderate Depression | 126 | 11.86 | 1.48 | 0.017 | -0.158, 0.192 |
| Moderately Severe Depression | 38 | 16.45 | 1.52 | 0.048 | -0.276, 0.362 |
| Severe Depression | 11 | 21.73 | 2.33 | 0.002 | -0.599, 0.601 |
| No/Minimal Anxiety | 198 | 2.61 | 1.25 | 0.041 | -0.099, 0.180 |
| Mild Anxiety | 204 | 6.81 | 1.42 | -0.023 | -0.160, 0.115 |
| Moderate Anxiety | 70 | 11.39 | 1.31 | 0.052 | -0.186, 0.283 |
| Severe Anxiety | 18 | 16.61 | 1.75 | -0.024 | -0.486, 0.448 |

*Correlation significant at 0.05 level, ** Correlation significant at 0.001 level
